# Helsmoortel–van der Aa syndrome in a Chinese pediatric patient due to *ADNP* nonsense mutation: A case report

**DOI:** 10.3389/fped.2023.1122513

**Published:** 2023-03-30

**Authors:** Li-juan Chen, Zhong-min You, Wen-hong Chen, Si Yang, Chun-chen Feng, Hai-yong Wang, Ting Wang, Yuan-yuan Zhu

**Affiliations:** ^1^ Department of Pediatric Neurology, Maternal and Child Health Hospital of Hubei Province, Wuhan, China; ^2^ Department of Marketing, Aegicare (Shenzhen) Technology Co., Ltd., Shenzhen, China

**Keywords:** Helsmoortel–van der Aa syndrome, ADNP, developmental delay, autism spectrum disorder, case report

## Abstract

**Background:**

Helsmoortel–van der Aa syndrome, also known as *ADNP* syndrome, is a condition that causes developmental delay, language impairment, autism spectrum, and variable extraneurologic features. It is caused by heterozygous mutations in the *ADNP* gene on chromosome 20q13. Most of the genetic causes of Helsmoortel–van der Aa syndrome have been reported are as *de novo* nonsense or frameshift stop mutations in exon 5 of *ADNP* gene, while fewer truncating variants were discovered in exons 4 and the 5′ end of exon 5.

**Methods:**

In our study, a 4-year-old female Chinese patient was reported with delayed psychomotor development, language impairment, ataxia, anxiety, aggressive behavior, and congenital heart defect. Trio whole exome sequencing and copy number variation sequencing were performed.

**Results:**

A novel *de novo* heterozygous pathogenic mutation c.568C > T (p.Gln190Ter) was identified in the *ADNP* gene of the proband. His unaffected parents did not have the variant. According to the American College of Medical Genetics (ACMG) guidelines, c.568C > T was classiﬁed as “pathogenic”.

**Conclusion:**

Our report indicated that c.568C > T (p.Gln190Ter) in *ADNP* gene is the cause of abnormal development of the nervous system, congenital heart disease and strabismus, broadening the spectrum of *ADNP* gene mutations associated with Helsmoortel–van der Aa syndrome.

## Introduction

Helsmoortel–van der Aa syndrome (HVDAS), is characterized by global developmental delay (GDD)/intellectual disability (ID), language impairment, autism spectrum, and variable extraneurologic features (1, 2). HVDAS (also called *ADNP* syndrome) usually occurs in infancy and is caused by heterozygous *de novo* mutations in the Activity-Dependent Neuroprotective Protein (*ADNP*) gene located on chromosome 20q13 (3). The ADNP protein plays a crucial role in the brain formation by regulating the expression of many other genes (4–6). Functional investigation of mice indicates that *ADNP* gene haploinsufficiency results in cognitive and social deficiencies (5, 7). In this study, we describe a female pediatric patient, with mild cognitive impairment, language development delays, abnormal gait, behavioral abnormalities, and congenital heart defect (CHD). A novel *de novo ADNP* nonsense mutation was identified.

## Methods

### Whole exome sequencing

Genomic DNA samples were extracted from whole blood using RelaxGene Blood DNA System (Tiangen, Beijing, China). Quality of genomic DNA was evaluated by Qubit3.0 and agarose gel analysis. DNA was sheared into proper pieces (150–200 bp) by a Covaries ultrasonicator. The target genomic regions were captured by hybridizing the genomic DNA sample library with the xGen® Exome Research Panel v1.0 (IDT, United States). High-throughput sequencing was then performed Illumina NovaSeq6000 (Illumina, San Diego, CA, United States) with 150 base-paired end reads. Data analysis methods refer to previous literature (8).

Sanger sequencing was arranged to confirm the mutations identified by trio WES. Primers (F: GCATCAAGGGTTTGGATCGG; R: TTTCTGCTGCAGCGCTTGTC) were designed by Primer3. PCR was conducted with TaKaRa Taq DNA Polymerase and premix under the following conditions: initial denaturation at 95°C for 5 min, followed by 32 cycles at 95°C for 30 s, 58°C for 30 s and 72°C for 40 s and a final hold at 72°C for 10 min. PCR products were purified and sequenced using an ABI 3730 DNA Analyzer with the BigDye™ Terminator Cycle Sequencing Kit (Applied Biosystems, Foster, CA, United States).

### CNV sequencing

50 ng of amniocyte DNA was fragmented and DNA libraries constructed by end filling, adapter ligation, and PCR amplification. DNA libraries were subjected to massively parallel sequencing on Illumina NovaSeq6000 (Illumina, San Diego, CA, United States) with 150 base-paired end reads. Using the GRCh37 genomic sequence as reference, a total of 30 million reads were precisely mapped using the Burrowse-Wheeler algorithm (9). Mapped reads were allocated progressively to 25 kilobase (kb) bin sizes from the *p* to *q* arms of the 24 chromosomes. Counts in each bin were then compared between all test samples run in the same flow cell to evaluate copy number changes using Weaver algorithms (10).

## Results

**Figure 1:**
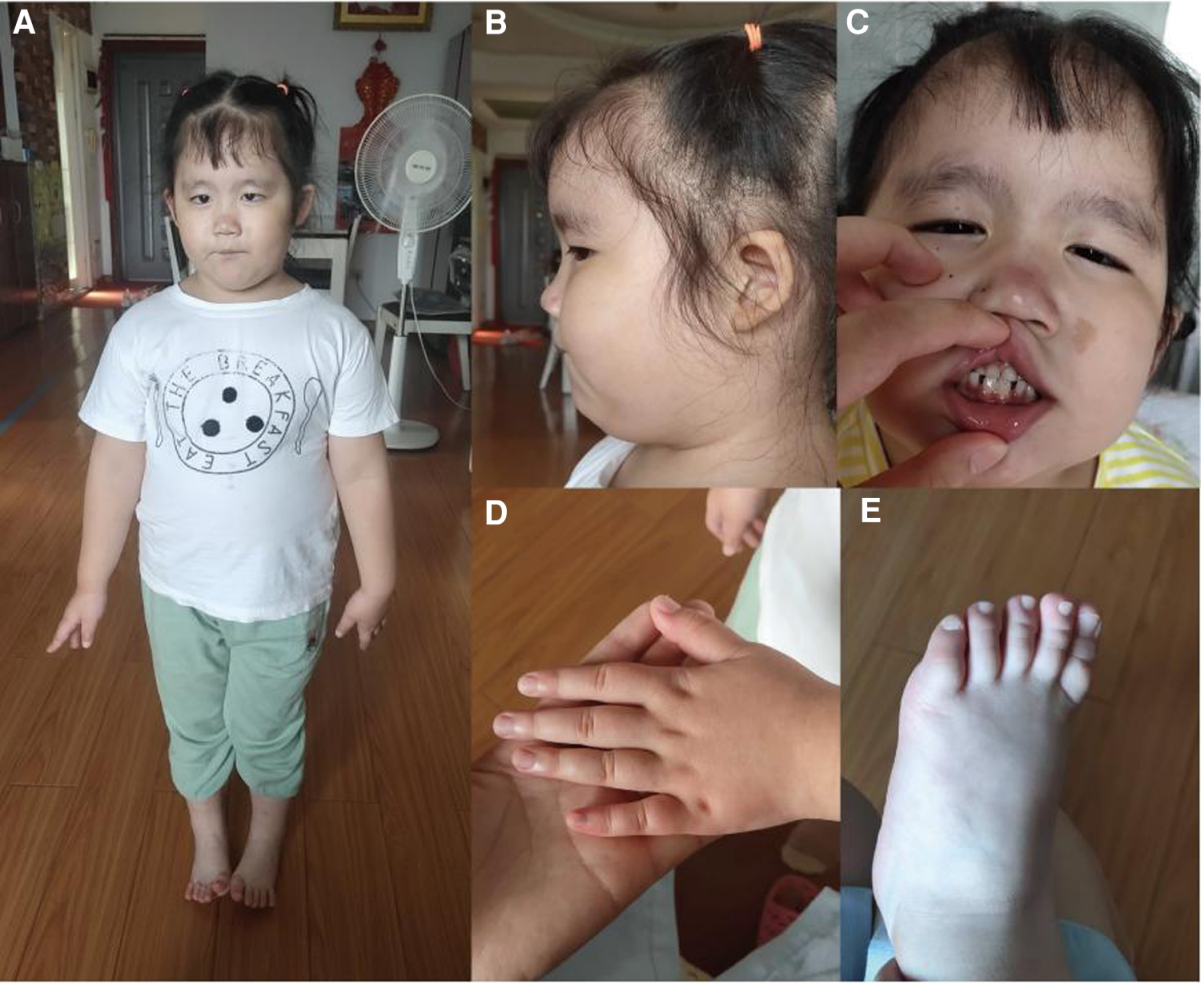
(**A,B**) Fat, short and thick neck, dysplasia of the both auricles, low-set ears; (**C**) Café au lait spot on face, a bit broad nasal bridge and teeth sparse; (**D,E**) short hands and feet.

A 4-year-old female child (G3P1), born to non-consanguineous parents, presented to the pediatric neurology department with mild cognitive impairment, language development delay, abnormal gait, and behavioral abnormalities (including fear, anxiety, and aggressive behavior). The mother had two adverse pregnancy outcomes due to unknown reasons. The third pregnancy went well and prenatal tests were normal. The patient was born at full-term by a caesarean section, with a birth weight of 3,900 g. Routine neonatal examination revealed atrial septal defect. Surgical repair of atrial septal defect was carried out when she was 3 years old. After the operation, the patient's heart was in good condition and no residual shunting was found by Doppler ultrasonographic examination. She started walking without support at 18 months. The patient had a normal range of motion but she had poor control of her limbs and abnormal gait. She was able to communicate verbally but not fluently, accompanied with fear, anxiety, and aggressive behavior when she appeared in public. She was unable to engage in active social behavior. She had a Café au Lait Spot on face, dysplasia and low-set ears, broad nasal bridge, strabismus, short and thick neck, and short upper limbs and fingers (Figure 1). She also showed deformation of the fingers, likely due to her actions of frequently biting or chewing on fingers. Like most other HVDAS patients, she had a gastrointestinal problem, which is constipation (11). And she had premature teething, which began when she was three months old and was almost full erupted dentition by 1 year of age (7). Electroencephalogram showed slightly slow background rhythm, increased activity of background slow waves, and tiny spinous and sharp waves were found in the right frontal region during wakefulness. Parents and caretakers feel that the child is intellectually and socially inferior to their peers and lack complete self-care ability. Sensory integration training in special education centers to improve language skills and cognitive abilities. Trio whole exome sequencing (trio WES) and copy number variation sequencing were performed on blood samples to detect a potential genetic abnormality.

**Figure 2:**
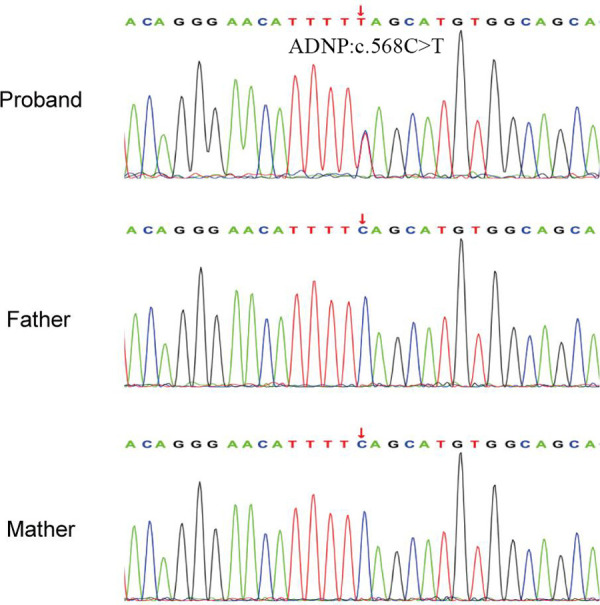
Sanger sequencing results of the pedigree. The proband had a heterozygous variant of c.568C > T in ADNP gene.

**Figure 3:**
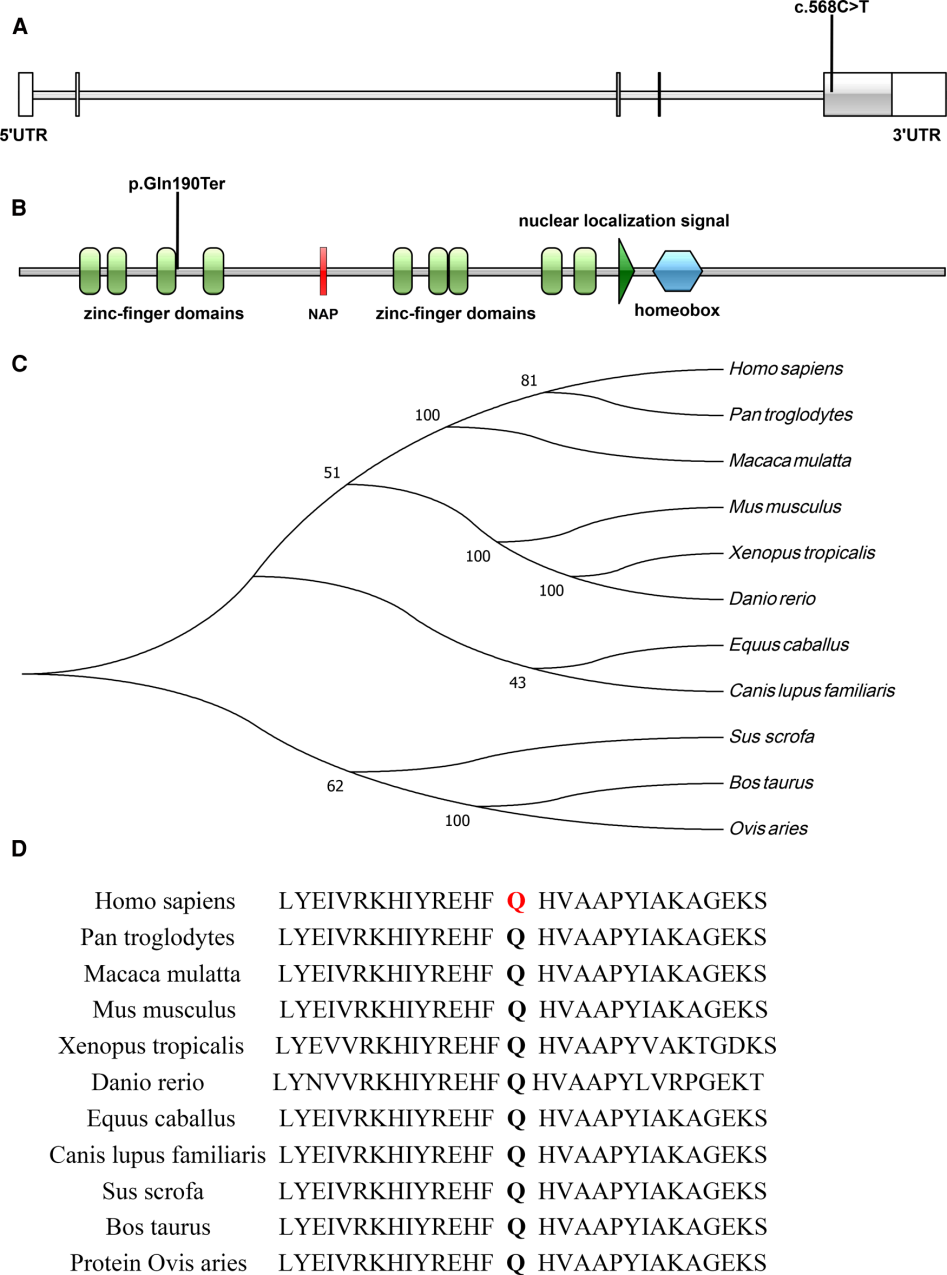
(**A,B**) Illustrations denoting the mutant sites, (**A**), nucleotide sequence; (**B**), protein sequence; (**C**) evolutionary relationships of species; (**D**) amino acid conservation in mutant sites.

A *de novo* variant in *ADNP* gene (c.568C > T, p.Gln190Ter) in exon 5 was discovered in the patient, which leads to premature termination of the gene coding process (Figures 3A,B). The variant was verified in the patient, but not found in other family members by Sanger sequencing (Figure 2). No abnormalities were found as a result of the CNV sequencing.

**Figure 4:**
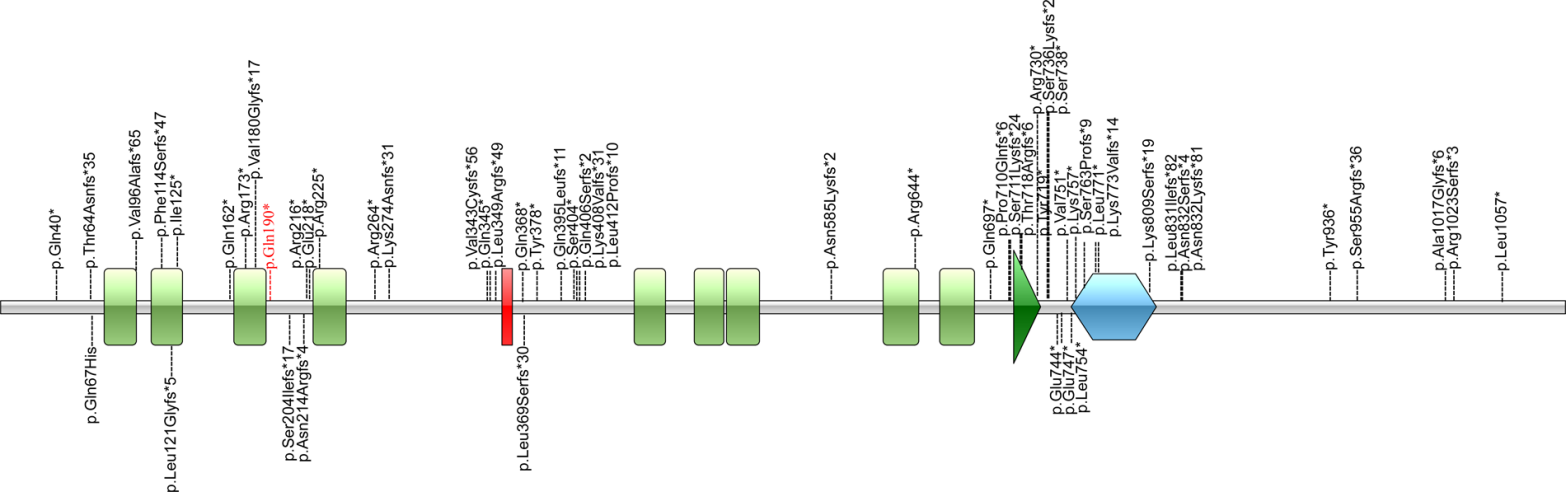
Illustrations denoting the mutant sites. Previously found varients in patients with HVDAS are depicted in black. The variants found in our patient p. Gln190* in red is the novel variant.

The c.568C > T (p.Gln190Ter) mutation is located in exon 5 of the *ADNP* gene and causes incorporation of a premature stop codon in the transcript, thereby truncating the protein (Figures 3A,B, 4). The variant c.568C > T was not found in gnomAD, EXAC, 1000genomes, or ESP6500. Pathogenic computational verdict was made based on pathogenic predictions from LRT (0.00), MutationTaster (1), FATHMM_MKL (0.984), DANN (0.998), EIGEN (1.102). The software (GERP++: 6.08, phyloP: 9.585, phastCons: 1.000 and SiPhy: 20.663) predicted the site to be highly conserved. The conservative analysis indicated that the glutamate residue at position 190 was located in a highly evolutionary-conserved region (Figures 3C,D). Therefore, c.568C > T in *ADNP* gene is classified as “pathogenic” according to American College of Medical Genetics guidelines (12, 13).

## Discussion

Helsmoortel–van der Aa syndrome is a condition that causes developmental delay, language impairment, autism spectrum, and variable extraneurologic features. It is caused by heterozygous mutations in the *ADNP* gene. Most of reported pathogenic variants of *ADNP* gene are premature stop mutations in the 3′ end of exon 5, while N-terminal variants are less reported. In our study, a novel pathogenic mutation c.568C > T (p.Gln190Ter) was identified in the *ADNP* gene, thus leading to partial protein degradation.

*ADNP* gene consists five exons and encodes 1102 amino acids (NM_015339.5). The first two exons are out of the coding region. Exon 5 is the largest exon, accounts for 94% of the coding sequences. Nine zinc-finger domains and one homeobox are also encoded by exon 5 (3, 14). Over sixty variants classified as “likely pathogenic” or “pathogenic” have been reported in ClinVar database, most of which are stop mutations (nonsense or frameshift) in exon 5. ADNP, is a neuroprotective protein that contains nine zinc-finger domains, one homeobox, and a nuclear localization signal (from residue 714 to 733) (3). As a transcription factor, ADNP is involved in neuronal cell differentiation and maturation by regulating the expression of a series of genes (4–6). Complete deficiency of *Adnp* results in neural tube closure defects, leading to death in the first trimester (4, 15). Deficiency of *Adnp* in mouse model leads to significant aberrations in neurite numbers (16–18). Animal experiments and clinical evaluations have confirmed that *Adnp* haploinsufficiency is associated with cognitive and social deficiencies (5, 7, 19, 20).

The distribution of these mutations is not random. Fewer mutations were discovered in the first half of exon 5 compared to the second half of exon 5. Correlations between the locations of truncation mutations and their impact on protein function were studied. Cappuyns et al. demonstrated (21) that different mutations in *ADNP* gene showed different patterns of expression and subcellular localization. N-terminal truncated protein before the fifth zinc finger (up to Residue 447) were routed towards cytosolic proteasomal degradation. Mutations spanning from Residue 473 up to 719 were observed to be cytoplasmic, whereas C-terminus mutants (from Residue 730 on) were imported to the nuclear. This is due to the fact that there is a bipartite nuclear localization signal from residue 714 to 733. There is a P*V*L motif at the downstream of the homeobox domain (Residue 754–814), which can bind HP1 protein. The homeobox domain and the P*V*L motif are the important functional structural units for the transcriptional activity of ADNP (4). Helsmoortel et al. (14) performed expression analysis of mutations (p.Lys831Ilefs*81 and p.Asp832Lysfs*80) in the P*V*L motif region and found that mRNAs transcribed from the mutant alleles significantly increased. They concluded that the upregulation of mutant mRNA might be due to the inability of the truncated protein to bind the *ADNP* promoter. In the study of Aboonq et al. (22), *ADNP* was a negative feedback autoregulatory gene, which means the wild-type ADNP protein could bind to the *ADNP* promoter and could repress *ADNP* gene expression.

In our case, c.568C > T (p.Gln190Ter) locates at the upstream of exon 5 in *ADNP*, between the third and fourth zinc finger domains. According Cappuyns et al. (21), p.Gln190Ter is near the N-terminal and may thus lead to partial protein degradation. The impact on the ADNP protein caused by p.Gln190Ter warrants further study.

Levine et al. reported a case of a man with severe phenotype, who was detected to carry a mutation of c.537dupA, p.Val180Serfs*2, which is similar to the mutation location in our reported case (23). We think it might be a gender difference. In the mouse model, Malishkevich et al. found that the expression of *ADNP* in the hippocampus of male mice was doubled compared with that of female mice, but in the case of haploid deficiency, the expression of *ADNP* in the hippocampus of male mice showed a two-fold decrease, while that in female mice was unchanged (24). The same sex difference was observed in songbirds, with higher levels of *ADNP* mRNA in the brains of males (25). In the study of Helsmoortel et al. (14), two female patients with the same recent mutation showed mild intellectual disability, and 5/6 of all reported male patients had severe intellectual disability. Sex-dependent expression of *ADNP*, which regulates over 400 genes essential for brain formation/organ development, may be responsible for differences in phenotype between male and female patients (4–6, 26). *Adnp* sex association may also be related to the fact that *ADNP* regulates sex steroid biosynthesis (27). Even in women with similar mutations, there are some phenotypic differences among them. Unlike the study of Helsmoortel, our patient showed no feeding problems and had constipation (14).

According to a recent study, the location of the *ADNP* gene mutation corresponds to different methylation pattern. In our case, the mutation is located at the N-terminal and associated with Class I episignatures (28, 29), which may also be responsible for the mild phenotype of the patient. An interesting fact is that most children with early tooth eruption present the epi-*ADNP*-1 signature (7), Breen et al. found that the two mutational groups differ in the mean age of first walking and rates of ASD (29). These results suggest that the correlation between methylation signatures and phenotypes are present but not particularly clear.

The Patient in our case presented with mild delayed psychomotor development, language impairment, ataxia, anxiety, aggressive behavior, and CHD. Up to 5-year-old, language and motor function of the patient improved after rehabilitation training. She still unable to actively participate in social activities, but passive social behaviors could be accomplished. In most of the cases reported previously, autistic traits were described. In our case, the patient has mild social impairment and no repetitive behavior was observed. According to Arnett et al. (30), verbal skill was the key explanation of individual variability in social impairment and the level of social difficulties in HVDAS caused by *ADNP* gene mutations was associated with the level of verbal impairment, consistent with our observation of residual verbal communication and thus mild social impairment in the patient.

## Conclusion

In summary, we reported a patient with mild developmental delay, mild social impairment, and CHD. She has a *de novo* heterozygous *ADNP* nonsense mutation in the 5′ end of exon 5, where few pathogenic mutations had been reported previously.

## Data Availability

The original contributions presented in the study are included in the article/Supplementary Material, further inquiries can be directed to the corresponding author.
